# Supplementary material for: Association between cerebral oximetry and return of spontaneous circulation following cardiac arrest: A systematic review and meta-analysis
Source: PLoS One. 2020 Aug 28;15(8):e0234979. doi: 10.1371/journal.pone.0234979 (PMC7454961; doi:10.1371/journal.pone.0234979)
Supplement: S1 Flow diagram — (DOC) [file pone.0234979.s002.doc]

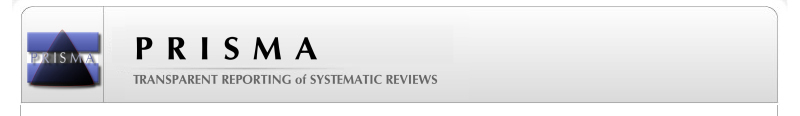
**PRISMA 2009 Flow Diagram**

**Screening**

**Included**

**Eligibility**

**Identification**

Records identified through database searching
(n = 201 )

Additional records identified through other sources
(n =0 )

Records after duplicates removed
(n = 162 )

Records screened
(n = 39 )

Records excluded
(n = 0 )

Full-text articles assessed for eligibility
(n =39 )

Full-text articles excluded, with reasons
(n = 26 )

Studies included in qualitative synthesis
(n = 13 )

Studies included in quantitative synthesis (meta-analysis)
(n = 13 )
