# Supplementary material for: Association between cerebral oximetry and return of spontaneous circulation following cardiac arrest: A systematic review and meta-analysis
Source: PLoS One. 2020 Aug 28;15(8):e0234979. doi: 10.1371/journal.pone.0234979 (PMC7454961; doi:10.1371/journal.pone.0234979)
Supplement: Attachment — Submitted filename: Response letter.docx [file pone.0234979.s003.docx]

**Manuscript ID:** PONE-D-19-30622

**Title:** Association between cerebral oximetry and return of spontaneous circulation following cardiac arrest: a systematic review and meta-analysis

**Journal:** PLOS ONE

**Response to Reviewers' comments**

Dear Editor,

We thank you for your careful consideration of our manuscript. We appreciate your response and overall positive initial feedback and made modifications to improve the manuscript. After carefully reviewing the comments made by the Reviewers, we have modified the manuscript to improve the presentation of our results and their discussion, therefore providing a complete context for the research that may be of interest to your readers.

We hope that you will find the revised paper suitable for publication, and we look forward to contributing to your journal. Please do not hesitate to contact us with other questions or concerns regarding the manuscript.

Best regards,

**Reviewer #1**

*In the abstract the authors write in the first sentence that the initial and mean rSO2 play a role in the efficiency of CPR. However it is impossible that an initial value as also the mean values, which is generated posthoc can influence the quality of CPR. The authors should adapt this sentence.*

**Response:** We are sorry for this. We meant "regional cerebral oxygen saturation (rSO2)".

*What do the authors mean with patients status in the abstract and further in the manuscript? In the Tables it is the location of the cardiac arrest. The authors should indicate this in the abstract and the first time they mention this in the manuscript.*

**Response:** The Reviewer is right. It was corrected accordingly.

*The authors make a difference between OHCA and IHCA patients however they should also investigate the moment of measurements. As in some studies, cerebral oxygenation is measured pre-hospital, sometimes at the ED of OHCA, sometimes In hospital at the place of arrest or at the ED once the IHCA patient is brought to the ED. You can expect differences in saturation values as the time between cardiac arrest and start of measurements is different. Do the authors analyze the data categorized following the moment of measurement OH or IH?*

**Response:** We thank the Reviewer for the comment. As this was a meta-analysis, we had to work with the data available from the previous papers. As such, unfortunately, we could not perform the analysis suggested by the Reviewer.

*The authors write that cardiac arrest is a disease however they should adapt this as it is an acute endpoint of many diseases.*

**Response:** We agree with the Reviewer. It was edited as suggested.

*Line 41: the authors give a survival rate of 6,3% however they don't give any reference. Can they add a reference?*

**Response:** We thank the reviewer for this kind reminding, a reference was added.

*Line 52: this sentence should be adapted as none of the other studies until today could associate rSO2 and neurological outcome.*

**Response:** We agree with the Reviewer. It was edited.

*Line 53: rSO2 is not widely used pre-operative.*

**Response:** We agree with the Reviewer. It was edited.

*Line 59: what do they mean with specific characteristics?*

**Response:** We thank the Reviewer. We meant patients with different characteristics. It was corrected.

*The authors should also search under near infra red spectroscopy.*

**Response:** We thank the Reviewer. This keyword was not included in our original protocol. Nevertheless, we made verification, and it did not yield additional eligible studies.

*How and why was decided to use initial and mean rSO2 values?*

**Response:** The present study was a meta-analysis of published studies. We used the data available from those studies.

*Are the authors sure they included all manuscript about cerebral saturation and cardiac arrest during they research period? A recent study was published by Genbrugge et al. using cerebral oximetry in CA patients. Why was this study excluded? Why are the studies of Ito et al. excluded?*

**Response:** We thank the Reviewer for the comment. We performed the search in May 2018. The study by Genbrugge et al. was published in August 2018. The studies by Ito et al. were not included because they did not match the inclusion criteria.

*On what is the subgroup analysis based?*

**Response:** We thank the Reviewer for the comment. Subgroup analyses are often used in meta-analyses in order to determine whether some factors (e.g., publication year, study type, characteristics of the patients, etc.) could influence the results.

*Did the authors also investigated the different devices used to measure rSO2? As some devices measure until 0 other have a higher cut off point. This can influence in the calculated mean values and initial values as some devices for example indicate 15 for all values below 15*

**Response:** We thank the Reviewer for the comment. Unfortunately, the number of different devices was too large, and the number of studies for some devices was too small for a reliable analysis. This will have to be examined in a future study with a different study design to try to include as many papers as possible for each device. This was included as a limitation.

**Reviewer #2**

*-All results should be separated based on prospective vs. retrospective and in-hospital vs out-of-hospital throughout the entire paper as it is not ideal to perform statistics combining these groups*

**Response:** We agree with the Reviewer that combining the two types of studies is not ideal, but it is a limitation of many meta-analyses. Nevertheless, the results of the meta-regression (Table 3) show that the study type did not influence the outcome of the meta-analysis. Perhaps when additional studies have been published, we will be able to perform a meta-analysis specifically for prospective and retrospective studies. This is already included in the Limitations.

*-In your methods, describe what the term 'patient status' means, as this is a very general term*

**Response:** We thank the Reviewer. We meant "the location of the cardiac arrest". It was clarified throughout the manuscript.

*-More detail is needed in the statistical analysis section to describe what you mean by 'subgroup analysis' and 'meta-regression analysis' as these terms alone don't convey what you are specifically addressing with these analyses (i.e. what outcomes are you looking at)*

**Response:** We thank the Reviewer. Subgroup analyses and meta-regression were performed to determine whether the characteristics of the studies and populations of patients influenced the conclusion of the meta-analysis [1]. The subgroup analyses were based on the publication year, study design, mean age, the percentage of males, the location of cardiac arrest, and study quality.

*-Instead of saying the relationships can be biased by 'percentage of males', it would be better to state they are influenced by gender. Additionally, if gender does influence the results, it would be beneficial to add a table that specifically separates results for males and females if this data is available.*

**Response:** We thank the Reviewer. The statement was edited accordingly. As for the data, we could not extract the separate data by sex from the included studies.

**References**

1. Thompson SG, Higgins JP (2002) How should meta-regression analyses be undertaken and interpreted? Stat Med 21: 1559-1573.
