# Supplementary material for: Association between cerebral oximetry and return of spontaneous circulation following cardiac arrest: A systematic review and meta-analysis
Source: PLoS One. 2020 Aug 28;15(8):e0234979. doi: 10.1371/journal.pone.0234979 (PMC7454961; doi:10.1371/journal.pone.0234979)
Supplement: Attachment — Submitted filename: Response letter.docx [file pone.0234979.s004.docx]

**Manuscript ID:** PONE-D-19-30622R3

**Title:** Association between cerebral oximetry and return of spontaneous circulation following cardiac arrest: a systematic review and meta-analysis

**Journal:** PLOS ONE

**Response to Reviewers' comments**

Dear Editor,

We thank you for your careful consideration of our manuscript. We appreciate your response and overall positive feedback and made modifications to improve the manuscript.

We hope that you will find the revised paper suitable for publication, and we look forward to contributing to your journal. Please do not hesitate to contact us with other questions or concerns regarding the manuscript.

Best regards,

**Reviewer #1**

*In line 213 and further the authors added two sentences using a reference that is not about cardiac arrest patients. They should delete these sentences as they don't have have an added value for the discussion.*

**Response:** We thank the Reviewer for the comment. Those two sentences were deleted.

*The authors replied that they added the study of Genbrugge et al. in the discussion and not in the analyses as it was published outside the study period. However the study period was until may 2018 and the manuscript was published in March 2018. As the number of included patients in that study is quiet big I think this is a methodological mistake of the authors if it was not included initially and I understand that it is a lot of work to redo the analyses. However at least they can add this to the disccusion as also the manuscript. The authors replied they added this manuscript to the disccusion however I don't find this. So can they pleas adjust this?*

*(Resuscitation. 2018 Aug;129:107-113. doi: 10.1016/j.resuscitation.2018.03.031. Epub 2018 MAR23 (!!!!) .Cerebral saturation in cardiac arrest patients measured with near-infrared technology during pre-hospital advanced life support. Results from Copernicus I cohort study. Genbrugge C1, De Deyne C2, Eertmans W3, Anseeuw K4, Voet D5, Mertens I6, Sabbe M7, Stroobants J8, Bruckers L9, Mesotten D10, Jans F11, Boer W12, Dens J13.)*

**Response:** Again, we thank the Reviewer for the comment. We agree that the epub date is before May 2018, but the actual publication was in August 2018. Because access to the Internet is not as free and easy in China as it can be in other countries, there might be a lag in access to the latest data, and we might have to wait for the paper versions, in some cases. We agree that it would add much to our meta-analysis in terms of patient numbers, but we added it in the Discussion.
